# Verification of Three-Phase Dependency Analysis Bayesian Network Learning Method for Maize Carotenoid Gene Mining

**DOI:** 10.1155/2017/1813494

**Published:** 2017-07-30

**Authors:** Jianxiao Liu, Zonglin Tian

**Affiliations:** ^1^College of Informatics, Huazhong Agricultural University, Wuhan 430072, China; ^2^National Key Laboratory of Crop Genetic Improvement, Huazhong Agricultural University, Wuhan 430072, China; ^3^School of Computer Science & Engineering, Northeastern University, Shenyang 110000, China

## Abstract

**Background and Objective:**

Mining the genes related to maize carotenoid components is important to improve the carotenoid content and the quality of maize.

**Methods:**

On the basis of using the entropy estimation method with Gaussian kernel probability density estimator, we use the three-phase dependency analysis (TPDA) Bayesian network structure learning method to construct the network of maize gene and carotenoid components traits.

**Results:**

In the case of using two discretization methods and setting different discretization values, we compare the learning effect and efficiency of 10 kinds of Bayesian network structure learning methods. The method is verified and analyzed on the maize dataset of global germplasm collection with 527 elite inbred lines.

**Conclusions:**

The result confirmed the effectiveness of the TPDA method, which outperforms significantly another 9 kinds of Bayesian network learning methods. It is an efficient method of mining genes for maize carotenoid components traits. The parameters obtained by experiments will help carry out practical gene mining effectively in the future.

## 1. Background

Vitamin A plays critical roles in many physiological processes of organisms, such as immune functions. About 250,000–500,000 children in the world suffer from blindness each year owing to vitamin A deficiency [1], which is an urgent problem to be solved at present. Maize is one of the crops that are rich in vitamin A. The carotenoid components (such as alpha-carotene, beta-carotene, and beta-cryptoxanthin) can be converted into vitamin A in the human body. Mining the genes related to maize carotenoid components and improving the content of vitamin A through genomic methods are some of the economic and effective ways to solve the problem of vitamin A deficiency.

It is well known that the quality of vitamin A (such as carotenoids) is related to the variation of the DNA sequence (including the epigenetic variation). DNA sequence variation is usually caused by affecting gene transcription (including transcription or not and the level of transcriptional expression), protein translation, and metabolites synthesis or degradation. Ultimately, we can see the visible phenotypic changes. Gene transcription is one of the key steps in the phenotype variation. In recent years, with the mature and rapid development of many high throughput technologies, several types of biological data are produced through biological experiments, including the data of genomics, transcriptome, and phenotypic. How to use the bioinformatics methods to mine the genes for carotenoid components from these massive data is important to improve the carotenoid content and the quality of maize.

## 2. Related Work and Our Approach

### 2.1. Related Work

(*1) Linkage Analysis and Genome-Wide Association Analysis (GWAS). *The linkage analysis method has been widely used to locate the genetic loci of the carotenoid components in maize. But the accuracy of this method is not high, generally between 10~30 cM, and QTL fine mapping is quite time-consuming. Later the genome-wide association analysis has become an important method for gene mining. Owens et al. have used this method to locate the genes about the synthesis and maintenance of carotenoids [2]. But this method can only detect the correlation of the single locus and phenotypic traits at a time [3]. In recent years, expression Quantitative Trait Loci (eQTL) was also used for gene location. Fu et al. have found 55 genes that might be related to carotenoid biosynthesis [4].

(*2) Mathematical and Statistical Methods*. In recent years, some mathematical and statistical methods are used to study the genetic loci for specific phenotypic traits, such as using linear regression method for the detection of cancer sites [5], using structural equation model to detect the effects of body size and obesity in mice [6], using ordinal regression for the detection of multiple phenotypic loci [7], and using logistic regression to detect the interaction between SNP (Single Nucleotide Polymorphism) loci associated with diseases [8, 9]. Dimensionality Reduction Multifactor (MDR) can detect the interaction between multiple genetic loci and their association with phenotypic traits [10]. This method has been successfully used to study the genetic loci for complex diseases, such as breast cancer, cardiovascular, and diabetes mining [11, 12].

(*3) Bayesian Network and Other Machine Learning Methods*. At present, machine learning related methods are more and more used in the study of genetic loci about disease and some other complex phenotypic traits. Clustering is a widely used method, such as hierarchical clustering method [13]. Some research work uses support vector machine and random forest method, like using support vector machine for the cancer classification of gene selection [14], using the random forest for the mining of gene locus about elderly blindness diseases and detection of feature gene (Kursa et al. 2014), and so on. Other methods include combining decision trees and particle swarm optimization to select cancer related genes [15], as well as ant colony optimization method [16].

Bayesian method uses the prior knowledge and can realize the accurate computation, and it is more and more used in the research of gene locus mining, for example, using Bayesian theory to mine the disease associated loci [17], identifying pig nipple number related genes [18], detecting gene loci associated with breast cancer [19], and detecting the interaction between specific phenotypic traits loci (Zhang et al. 2007), [20]. Bayesian network is a probabilistic graphical model that represents a set of random variables and their conditional dependencies via a directed acyclic graph (DAG). It is more and more used in the research of gene locus mining. The Bayesian network structure learning method mainly includes the dependence analysis based method and search scoring based method. At present, the search scoring based method is mainly used in the gene mining research work. For example, using heuristic search of* k2* algorithm to construct the network of gene locus and autologous stem cell transplant disease [21], using heuristic search method to get the obesity-related genetic variants [22], mining the genes related to smoking disease using the minimum description length principle (MDL) scoring search method [12, 23], mining the genes related to wheat multiple quantitative trait using the Bayesian information criterion (BIC) scoring search method [24], discovering Alzheimer genetic biomarkers using tree augmented naive Bayes method [25], and using the scoring method for the detection of loci associated with human complex diseases (Han et al. 2013).

In the part above, we have elaborated several kinds of methods used for gene locus mining. These methods have different advantages and disadvantages, respectively, and we summarize them in Table 1.

### 2.2. Analysis and Our Approach

As has been described above, Bayesian network is an effective way for gene locus mining. The existing Bayesian network research work mainly uses the search scoring based method to construct the network of phenotypic traits and genes. But this method has the following two problems: (1) low learning efficiency and easy to cause local search. The search scoring based Bayesian network structure learning method often uses the local or random search strategy, and it is a combinatorial explosion problem with the increase of the node number. (2) Poor flexibility and low calculation accuracy. Most of the search score based methods are not flexible enough; for example, it cannot deal with the phenotypic traits as the root or leaf nodes in the network. This will increase the complexity of conditional independence judgement, thus affecting the conditional probability accuracy between phenotypic traits and gene nodes [26].

Compared with the search scoring based method, the efficiency of the dependence analysis based method is relatively high, and it can obtain the global and optimal solution. The three-phase dependency analysis algorithm (TPDA) is a commonly used dependence analysis based method [27]. This algorithm uses the global search mode, and it can quickly determine the correlation between nodes by computing the mutual information or conditional mutual information. Its learning efficiency is higher than the search scoring based method. In addition, the TPDA method uses the strategy of separating the conditional independence test and the network structure judgement. It needs to do a large number of conditional probability calculations, which is suitable for sparse graph processing. In this work, the correlation between maize carotenoid components and related genes is sparse [28, 29]. Therefore, we plan to use the three-phase dependency analysis Bayesian network structure learning method to construct the network including maize genes and carotenoid components. This algorithm uses the conditional mutual information and open path judgement to do the conditional independence test. It leads to the result being not reliable due to the high order conditional independence test. According to the normal distribution of the transcriptome data and phenotype data, we intend to use the entropy estimation approach of Gaussian kernel probability density estimator to calculate the mutual information between nodes [30]. This method can effectively solve the result unreliable problem caused by high order conditional independence test. In addition, we use 5 kinds of carotenoid component phenotypic traits and 4 reported genes of maize association population material to do the experiment verification and analysis. Experiment results show that the three-phase dependency analysis Bayesian network structure learning method can effectively mine the genes related to maize carotenoid component traits. It can provide the useful resource information for the genetic basis analysis of maize complex quantitative traits.

## 3. Methods

### 3.1. Bayesian Network

A complete Bayesian network includes the following three parts: nodes, edges between nodes, and the conditional probability of all the nodes.

**Definition 1 (Bayesian network).**

It is defined as a tuple of *B* = {*S*, *P*}.

- *S* = {*X*, *E*} denotes the structure of Bayesian network, and: *X* = {*x*_*i*_, 0 ≤ *i* ≤ num} denotes the nodes in *B*. *E* = {*x*_*i*_ → *x*_*j*_, 0 ≤ *i* ≤ num, 0 ≤ *j* ≤ num} denotes the edges between nodes.
- *P* = {*p*(*x*_*i*_∣*pa*(*x*_*i*_)), *x*_*i*_ ∈ *X*} denotes the conditional probability table (CPT) of all the nodes in *B*, and: *pa*(*x*_*i*_) = {*x*_*p*_, *x*_*p*_ → *x*_*i*_∧(*x*_*p*_ → *x*_*i*_) ∈ *E*∧*x*_*p*_ ∈ *X*∧*x*_*i*_ ∈ *X*}, *pa*(*x*_*i*_), denotes the parent node set of *x*_*i*_.

In the above definition, *p*(*x*_*i*_∣*pa*(*x*_*i*_)) expresses the conditional probability of *pa*(*x*_*i*_). The joint probability distribution is equal to the product of conditional probability, as shown in (1). In the equation, *pa*(*x*_*i*_) is the parent node set of *x*_*i*_.

(1) $$\begin{array}{c} p\left(\;X\;\right)=\underset{x_{i}\in X}{\prod }p\left(\;x_{i}\;|\;pa\left(\;x_{i}\;\right)\;\right). \end{array}$$

Bayesian network structure learning refers to finding a network with the best fit for the given data set. It includes the following three steps to construct a network.

(*1) Determine the Variables and Domain of the Variables*. It will determine the variables of all the nodes in *X* of Bayesian network *S* = {*X*, *E*}.

(*2) Structure Learning*. It will determine the dependency relationships between variables, and the directed acyclic graph is used to express the network structure.

(*3) Parameter Learning*. It will learn the distribution between variables and get the conditional probability table (CPT) of all the variables.

### 3.2. Three-Phase Dependency Analysis Algorithm

The three-phase dependency analysis algorithm (TPDA) mainly includes three steps:* Drafting*,* Thickening,* and* Thinning* [27]. The first stage is* Drafting. *The correlation degree between any two nodes is calculated through the mutual information computation. When the mutual information is greater than the threshold, it means there exists an edge between the corresponding nodes. The initial network will be constructed using the above method. The second stage is conditional mutual information judgement* (Thickening)*. It firstly finds the cut set* C* between two nodes when there is an open path between them. Then the conditional mutual information about the two nodes and* C* will be calculated, and it will judge whether it is conditionally independent or not. If it is not independent, the corresponding edge will be inserted into the graph. Then the network of* I-MAP* will be got. The third stage is* Thinning*. For each edge* e *in the graph, it will be removed temporarily. Then it finds the minimum cut set *C*_min_ between the nodes of* e* and judges whether they are conditional independent or not. If they are conditional independent,* e* will be deleted forever. Otherwise,* e* will be inserted into the network again, and get* P-MAP* finally.

### 3.3. Our Approach

 (*1) Discretization Processing*. In order to enhance the learning efficiency, it often needs to do discretization operation on the expression data. In this work, we mainly use two discretization methods:* Interval* and* Quantile*. We denote multivalue discretization result as *N*-value, like 2-value, 3-value, and so on.

(*2) Initial Bayesian Network Construction (Stage of Drafting)*. We regard genes and carotenoid component traits as nodes in the network and calculate the mutual information of each two nodes firstly, including the mutual information MI(ge_*i*_, ge_*j*_) between gene ge_*i*_ and ge_*j*_ and MI(ge_*i*_, ct_*k*_) between ge_*i*_ and component trait ct_*k*_. The edge of the node pair whose mutual information is greater than the threshold will be inserted into an edge set named *S*; then we sort all the node pairs in *S* according to the value of mutual information. Then all the node pairs in *S* are judged to see whether there exists an open path between the corresponding nodes or not. If an open path exists, the edge of the node pair will be inserted into another edge set named *R*. Otherwise, we will insert the corresponding edge into the graph, thus constructing the initial network.

The transcriptome and carotenoid component phenotype data are often subject to the normal distribution. In this work, we use the entropy estimation approach of Gaussian kernel probability density estimator to calculate the mutual information [30]. For example, we use (2) to calculate the mutual information MI(ge_*i*_, ct_*k*_) of gene ge_*i*_ and carotenoid component trait ct_*k*_.

(2) $$\begin{array}{c} MI\left(\;ge_{i},ct_{k}\;\right)=\frac{1}{2}log\frac{\left|C\left(ge_{i}\right)\right|\cdot \left|C\left(ct_{k}\right)\right|}{\left|C\left(ge_{i},ct_{k}\right)\right|}. \end{array}$$

In the equation,* C* represents the covariance matrix of the expression of ge_*i*_ and carotenoid component ct_*k*_ and |*C*| represents the determinant of the matrix* C*. We can calculate the mutual information MI(ge_*i*_, ge_*j*_) of gene ge_*i*_ and ge_*j*_ using the same approach. The distribution of the child node is conditionally depended on the combination of values of the parent nodes in the Bayesian network [26]. Regarding the carotenoid component traits as the leaf nodes will greatly reduce the conditional probability calculation accuracy, we treat the carotenoid component traits as the root node in this work.

(*3) Conditional Independence Test (Stage of Thickening)*. On the basis of constructing initial network, we judge the node pair of* R* in turn using the conditional independence test. From the aspects of nontransfer connection, serial (transfer) connection, and convergence connection, we get the minimum cut set cutset which can* D*-separate the node pair of* R* in turn. Then we use (3) to calculate the conditional mutual information between the nodes in which there exists an open path, thus judging whether the node pair is conditional independent or not. If the conditional mutual information is greater than the threshold, it means the condition is not independent. Then we connect the two nodes using directed edge and judge the next node pair in* R* in turn.

(3) CMIgei,ctk ∣ cutset=12log⁡Cgei,cutset·Cctk,cutsetCcutset·Cgei,ctk,cutset.

In (3), ge_*i*_ represents gene, ct_*k*_ represents carotenoid component trait, cutset represents the minimum cut set,* C* represents the covariance matrix of the gene expression and the phenotype data of component trait, and |*C*| represents the determinant of the matrix* C*.

(*4) Network Optimization (Stage of Thinning)*. It will check each edge* e* in the network to achieve the further optimization of the network. Supposing two nodes of* e* is (node_*i*_, node_*j*_), if there exists an open path which connects node_*i*_ and node_*j*_ except for* e*, then we remove* e* temporarily and find the minimum cut set that can* D*-separate node_*i*_ and node_*j*_. Then we use (3) to judge whether the node pair is conditionally independent or not. If it is independent, then we delete* e*.

Through the above 4 steps, we can construct the network of maize genes and carotenoid component phenotypic traits. The genes related to maize carotenoid component traits will be got and it can provide genetic resource information for the genetic basis analysis of maize complex quantitative traits.

## 4. Experiment

### 4.1. Dataset

We have assembled a global maize germplasm collection with 527 elite inbred lines (association mapping panel, AMP) released from the major temperate and tropical/subtropical breeding programs of China, International Maize and Wheat Improvement Center (CIMMYT), and the Germplasm Enhancement of Maize (GEM) project in the US, which were chosen to be the representative of maize genetic diversity and/or for their promise in maize improvement. All of the lines were previously assayed by the 50K Maize SNP array (commercially available from Illumina). Deep RNA sequencing was also performed on 368 of the 527 lines using kernels harvested 15 days after pollination (DAP) [4]. The dataset of our germplasm, transcriptome, and phenotype is shown in Table 2. All the dataset can be got through http://www.maizego.org/ and http://modem.hzau.edu.cn/ [31].

### 4.2. Results

The* bnlearn* is an R package for learning the graphical structure of Bayesian network, estimating the parameters and performing some useful Bayesian inference. This package provides a number of underlying libraries about Bayesian network learning, including structure learning, parameter learning, and inference. In addition, this package is completely free and its code is entirely open and with good scalability. This package does not support the three-stage dependency analysis algorithm. We implement this algorithm using R and denote it as TPDA. In addition, we use the other 9 kinds of Bayesian network learning methods (including* gs*,* hc*,* iamb*,* mmpc*,* rsmax*,* tabu*,* fastiamb*,* interiamb*, and* mmhc*) to construct the network, to compare with our TPDA method.

The linkage analysis and association analysis methods have been used to locate a plurality of Quantitative Trait Locus (QTL) about maize carotenoid component traits so far. The following 4 genes related to maize carotenoid component traits have been reported:* lcyE* (GRMZM2G012966),* crtRB1* (GRMZM2G152135),* PSY1* (GRMZM2G300348), and* CRTISO* (GRMZM2G108457) [2, 28, 32–35]. In addition, we randomly select 100 genes, and then together with *α*-carotene (AC), *β*-carotene (BC), Lutein (LUT), Zeaxanthin (ZEA), *β*-Cryptoxanthin (Bcry), and above 4 genes composed of 109 nodes, we do the experiment 10 times, and the 10 experiments data can be seen in the supplementary file (Gene100_1.csv~Gene100_10.csv) in Supplementary Material available online at https://doi.org/10.1155/2017/1813494.

As has been described in Section 3.3, we firstly do the discretization processing operation on the expression and phenotype data and further use the Bayesian network learning method to construct the network. We mainly use two kinds of discretization methods:* Interval* and* Quantile*. And we denote multivalue discretization result as *N*-value, like 2-value, 3-value, and so on. We compare the learning efficiency and accuracy of 10 kinds of Bayesian network learning methods (*gs*,* hc*,* iamb*,* mmpc*,* rsmax*,* tabu*,* fastiamb*,* interiamb*,* mmhc,* and TPDA) when using the discretization methods of* Interval* and* Quantile*. The whole experiment results are shown in the supplementary files (9 kinds of methods-Interval.csv, 9 kinds of methods-Quantile.csv, TPDA-Interval.csv, and TPDA-Quantile.csv).

(*1) Learning Effect Comparison of Different Thresholds about TPDA*. In the* Drafting* stage of TPDA, it needs to set the threshold of mutual information. In the stage of* Thickening* and* Thinning*, it also needs to set the threshold of conditional mutual information. In the case of using the discretization of 8-value and 2-value, we compare the learning effect of different thresholds using Random5 data about the discretization methods of* Interval* and* Quantile*, as seen in Tables 3 and 4. In the table, the form of (*X*,* Y*,* Z*) refers to the thresholds that are set in the three stages, respectively. A refers to the number of edges between 4 genes and 5 component traits. B refers to the number of edges between other 100 genes and 5 component traits. The ratio of 4 genes is calculated by A divided by 4, and the ratio of other 100 genes is calculated by B divided by 100.

It can be seen that the learning effect is different of the two discretization methods when setting different thresholds. The two discretization methods have better learning effect when setting the thresholds to (0.01, 0.01, and 0.01). The learning effect of 8-value discretization is better than 2-value when taking a specific threshold. In addition, we can see the threshold of* Drafting* stage has a great influence on the result. In the case of setting the threshold of* Drafting* to 0.02, the learning effect is significantly better than the case of setting the threshold to 0.025. In all, we can see the TPDA method has the best learning effect when setting the threshold of (0.01, 0.01, and 0.01) and use the 8-value discretization.

(*2) Learning Efficiency Comparison of Different Discretization Values*. In the case of using different discretization values of* Interval* and* Quantile* methods, we compare the learning efficiency of different thresholds about TPDA, as seen in Table 5. The learning time is got by calculating the average values of 10 experiments and measured in seconds.

**Figure 1:**
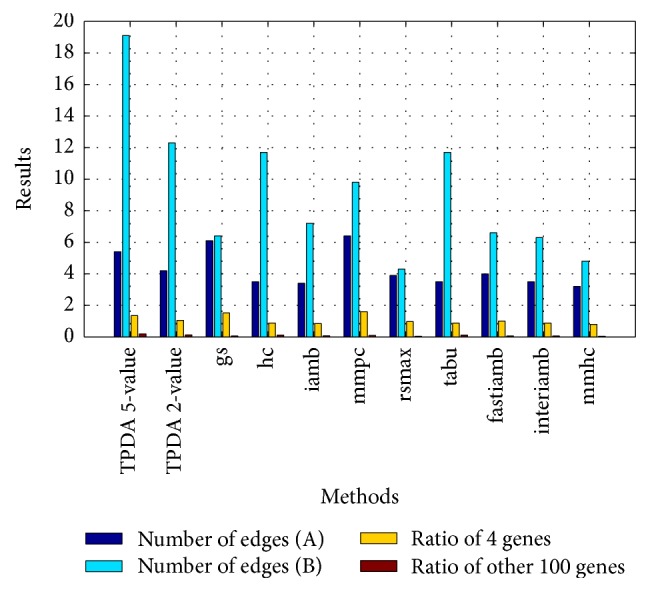
Learning result comparison of Interval discretization method.

**Figure 2:**
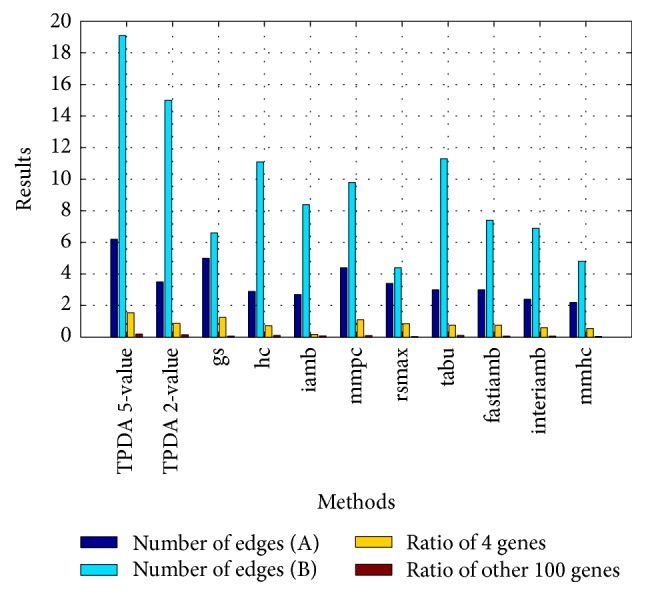
Learning result comparison of Quantile discretization method.

The learning efficiency of the* Quantile* discretization method is higher than the* Interval* method when taking particular threshold. The learning time of the two methods is becoming less as the threshold increases. In addition, we can see the learning time is becoming less as the discretization value becomes small. This is because the calculation times will be reduced as the discretization value becomes small.

(*3) Learning Results Comparison with Different Bayesian Network Learning Methods*. In this experiment, we compare the learning effect of different Bayesian network learning methods, and we set the threshold to (0.01, 0.01, and 0.01) of TPDA method. Experiment results show the other 9 kinds of Bayesian network learning methods have better learning effect in the case of using the 2-value discretization; therefore we use the 2-value discretization to do the comparison and analysis of the 9 kinds of methods. We calculate the average of 10 experiment results to ensure the accuracy. The learning results of* Interval* and* Quantile* discretization methods are shown in Figures 1 and 2, respectively. A refers to the average number of edges between 4 genes and 5 component traits. B refers to the average number of edges between other 100 genes and 5 component traits.

Through Figures 1 and 2, we can see the learning effect of the* Interval* discretization method is better than the* Quantile* discretization method on the whole for the 10 kinds of Bayesian network learning methods. For the TPDA method, the learning effect of 5-value is better than the case of 2-value. For the* Interval* discretization method, the learning effect of TPDA method is slightly worse than* gs* and* mmpc*, but it is better than the other 7 kinds of methods. For the* Quantile* discretization method, the learning effect of TPDA method is better than other 9 kinds of methods in the case of using the 5-value discretization. The learning effect of TPDA method is worse than* gs* and* mmpc* and better than the other 7 methods when taking the 2-value discretization. In all, we can see the TPDA method has better learning effect when using the* Quantile* and 5-value discretization method. And the learning effect of TPDA method is better than other 9 kinds of methods on the whole.

(*4) Learning Effect Comparison of Different Discretization Values*. In the case of setting different discretization values of* Interval* and* Quantile* methods, this experiment compares the learning effect of* gs*,* hc*,* iamb*,* mmpc*,* rsmax*,* tabu*,* fastiamb*,* interiamb*,* mmhc,* and our TPDA method. We compare the average value of 10 experiment results, and the learning effect is shown in Table 6. The threshold of TPDA is set to (0.01, 0.01, and 0.01). In the form of (*X*, *Y*), *X* refers to the average ratio of 4 genes about 10 experiments, and *Y* refers to average ratio of other 100 genes about 10 experiments.

We can see that the average ratios of about other 100 genes are greater than 0 in most cases; the reason is that there exist genes related to maize carotenoid content traits. It can be seen that the average ratio of 4 genes about TPDA method is larger than other 9 kinds of methods apparently when using specific *N*-value discretization method. The TPDA method has better learning effect of all the 10 methods. For the specific Bayesian network learning method, the value of *X* is larger than *Y*; it indicates that all the 10 kinds of methods can effectively mine genes related to maize carotenoid content traits. The other 9 kinds of Bayesian network learning methods have better learning effect when using 2-value discretization. When the discretization value is larger than 5, the other 9 kinds of methods cannot learn any effective edge basically. The TPDA method has better learning effect when using 7-value discretization approach. In addition, we can see the methods of* gs* and* mmpc* have better learning effect, and the learning effect of the other 7 kinds of methods is about the same.

(*5) Learning Efficiency Comparison of Different Bayesian Network Learning Methods*. In this experiment, we compare the learning time of* gs*,* hc*,* iamb*,* mmpc*,* rsmax*,* tabu*,* fastiamb*,* interiamb*,* mmhc,* and TPDA method, and it is measured in seconds. The results of the* Interval* and* Quantile* discretization method are shown in Tables 7 and 8, respectively. The threshold of TPDA is set to (0.01, 0.01, and 0.01), and the learning time is calculated by the average results of 10 experiments. When taking 8-value and 7-value* Interval* discretization, the methods of* gs*,* hc*,* iamb*,* mmpc*,* rsmax*,* tabu*,* fastiamb*,* interiamb,* and* mmhc* cannot learn any edges between 4 genes and 5 component traits. Therefore, we do not compute the learning time of 8-value, 7-value of Interval discretization in Table 7, and 8-value, 7-value, and 6-value of Quantile discretization in Table 8.

For the two discretization methods, the learning time of TPDA is much larger than other 9 kinds of methods. This is mainly due to it needing to do a large number of conditional independence judgements in the stage of* Thickening* and* Thinning*. The learning time of TPDA method is becoming less with the decrease of the discretization value. In addition, we can see the methods of* hc* and* fastimab* use the less time, and the methods of* gs*,* rsmas,* and* interimab* use the more time. This is consistent with the results that have been reported in the machine learning area.

On the whole, we can see the TPDA method performed better than other 9 kinds of Bayesian network learning methods. It can effectively mine the genes related to maize carotenoid component traits. In addition, experiment results show the TPDA method has the best learning effect when setting the threshold to (0.01, 0.01, and 0.01) and using the 7-value discretization. The other 9 kinds of Bayesian network learning methods have better learning effect when using the 2-value discretization approach. For the 10 kinds of Bayesian network learning methods, the learning effect of the* Interval* discretization method is better than the* Quantile* method on the whole. These obtained parameters will help carry out practical experiment applications effectively in the future, and thus help mine genes related to maize carotenoid component traits efficiently and accurately. It can also help mine the genes of specific traits for different species.

## 5. Conclusion

How to mine genes related to maize carotenoid components flexibly and efficiently is a key problem to be solved in the biology research. In this work, we use the three-phase dependency analysis algorithm (TPDA) Bayesian network structure learning method to construct the network of maize genes and carotenoid component traits, thus realizing gene mining for maize carotenoid components. The 5 kinds of carotenoid component traits and 4 reported genes about maize association population material are used to do the experiment validation and analysis. Experiment results show different methods have different learning effect and efficiency when setting different thresholds and discretization values. And our TPDA method performed better than other 9 kinds of Bayesian network learning methods. The parameters obtained by experiments will help carry out practical gene mining efficiently and accurately in the future. On the whole, experiment results show the three-phase dependency analysis Bayesian network structure learning method is an effective approach for mining the genes related to maize carotenoid component traits. The learning result can be used to provide genetic resources and useful information for genetic basis analysis of maize complex quantitative traits. It can also provide strong support for gene function mining and genetic breeding for maize.

## Supplementary Material

The experiment data 1 of 109 nodes, the experiment data 2 of 109 nodes, and so on are shown here.

## Figures and Tables

**Table 1:** The advantages and disadvantages of all the methods.

ID	Methods	Advantages and disadvantages
1	Linkage analysis method genome-wide association analysis	Advantages: detecting the relationship between genetic loci and phenotypic trait
		Disadvantages: low accuracy; time-consuming; high false positive rate; high cost

2	Expression Quantitative Trait Loci (eQTL)	Advantages: mining the relationship between genetic loci and quantitative trait
		Disadvantages: high false positive rate; high cost

3	Mathematical and statistical methods	Advantages: high efficiency; low cost
		Disadvantages: low accuracy; can not deal with the data with noise and nonlinear relationship; high computational complexity

4	Dimensionality Reduction Multifactor (MDR)	Advantages: detecting the epistasis interaction; low cost
		Disadvantages: high calculation complexity

5	Clustering method	Advantages: establishing the global relationship; low cost
		Disadvantages: cannot deal with data with nonlinear relationship; cannot detect the correlation between genes and phenotype

6	Support vector machine random forest method	Advantages: low cost; high efficiency
		Disadvantages: the correctness depends on the quality of training set, but the training set is often hard to obtain

7	Bayesian method	Advantages: using the prior knowledge; realize the accurate calculation; low cost
		Disadvantages: lack of network visibility; can not detect the epistasis interaction

8	Bayesian network	Advantages: processing data with noise and non-linear relationship; supporting different data types; high precision; low cost; detecting the epistasis interaction
		Disadvantages: low learning efficiency; easy to cause local search

**Table 2:** Our dataset.

Type	Data
Germplasm resources	527 inbred lines for association mapping panel (AMP) with different populations (143 lines for NSS, non-stiff-stock; 33 for SS, Stiff-stock; 232 for TST, tropical and semitropical; and the left 119 are regarded as MIXED)

Transcriptome quantification	28,769^a^ genes' quantitative expression of maize whole kernel (15 days after pollination, 15 DAP). Using Illumina high throughput sequencing technology, we get about 1 million 60 thousand high quality SNP markers and expression of 28,769 genes, which cover about 70% of the predicted genes in maize genome [4]

Phenotype data	5 kinds of carotenoid component phenotype data of 482 materials in association mapping panel: *α*-carotene (AC), *β*-carotene (BC), Lutein (LUT), Zeaxanthin (ZEA) and *β*-Cryptoxanthin (Bcry)

^a^Genes filtered as expressed in >50% lines.

**Table 3:** Results of different thresholds of Interval discretization method.

Results	Parameters									
	(0.01,0.01,0.01)		(0.015,0.015,0.015)		(0.02,0.02,0.02)		(0.025,0.025,0.025)		(0.02,0.01,0.01)	
	8-value	2-value	8-value	2-value	8-value	2-value	8-value	2-value	8-value	2-value
Number of edges (A)	7	6	4	3	2	2	3	1	4	2
Number of edges (B)	18	13	10	9	6	7	6	4	11	8
Ratio of 4 genes	1.75	1.53	1.0	0.75	0.5	0.5	0.75	0.25	1.0	0.5
Ratio of other 100 genes	0.18	0.1	0.1	0.09	0.06	0.07	0.06	0.04	0.11	0.08

**Table 4:** Results of different thresholds of Quantile discretization method.

Results	Parameters									
	(0.01,0.01, 0.01)		(0.015, 0.015,0.015)		(0.02, 0.02,0.02)		(0.025, 0.025,0.025)		(0.02, 0.01, 0.01)	
	8-value	2-value	8-value	2-value	8-value	2-value	8-value	2-value	8-value	2-value
Number of edges (A)	6	3	5	3	5	3	2	1	5	3
Number of edges (B)	16	14	13	9	10	9	9	7	14	12
Ratio of 4 genes	1.5	0.75	1.25	0.75	1.25	0.75	0.5	0.25	1.25	0.75
Ratio of other 100 genes	0.16	0.14	0.13	0.09	0.1	0.09	0.09	0.07	0.14	0.12

**Table 5:** Learning efficiency comparison of different thresholds and discretization values.

*N*-value	Parameters									
	(0.01,0.01, 0.01)		(0.015, 0.015,0.015)		(0.02, 0.02,0.02)		(0.025, 0.025,0.025)		(0.02, 0.01, 0.01)	
	Interval	Quantile	Interval	Quantile	Interval	Quantile	Interval	Quantile	Interval	Quantile
8-value	34.854	17.324	22.618	11.341	16.022	7.631	12.042	5.531	19.81	8.266
7-value	35.336	17.78	23.557	11.351	14.594	7.508	8.592	5.34	10.33	8.124
6-value	18.861	17.131	12.791	10.979	8.904	7.553	6.619	5.194	10.17	8.041
5-value	19.644	16.924	13.017	10.885	9.16	7.257	6.717	5.066	10.564	7.871
4-value	18.75	16.282	12.172	10.076	8.564	6.65	6.965	4.743	15.698	7.287
3-value	29.124	15.083	18.52	9.133	11.128	6.022	8.355	4.031	13.326	6.324
2-value	13.624	11.109	7.91	5.951	5.133	3.735	3.227	2.125	5.577	3.922

**Table 6:** Learning effect comparison of different discretization values.

Cases	Methods									
	TPDA	*gs*	*hc*	*iamb*	*mmpc*	*rsmax*	*tabu*	*fastiamb*	*interiamb*	*mmhc*
2-value (Interval)	(1.05,0.12)	(1.53,0.06)	(0,88,0.12)	(0.85,0.07)	(1.6,0.1)	(0.98,0.04)	(0.88,0.12)	(1.0,0.07)	(0.88,0.06)	(0.8,0.05)
3-value (Interval)	(1.38,0.17)	(0.0,0.05)	(0.25,0.05)	(0.05,0.08)	(0.35,0.09)	(0.0,0.04)	(0.25,0.05)	(0.4,0.04)	(0.05,0.07)	(0.0,0.04)
4-value (Interval)	(1.3,0.17)	(0.0,0.08)	(0.0,0.03)	(0.38,0.08)	(0.5,0.08)	(0.0,0.03)	(0.0,0.03)	(0.0,0.04)	(0.4,0.08)	(0.0,0.03)
5-value (Interval)	(1.35,0.19)	(0.0,0.04)	(0.0,0.02)	(0.0,0.04)	(0.0,0.04)	(0.0, .01)	(0.0,0.02)	(0.0,0.04)	(0.0,0.04)	(0.0,0.01)
6-value (Interval)	(1.5,0.19)	(0.0,0.02)	(0.0,0.01)	(0.0,0.04)	(0.5,0.03)	(0.0,0.0)	(0.0,0.01)	(0.0,0.04)	(0.0,0.04)	(0.0,0.0)
7-value (Interval)	(1.68,0.19)	(0.0,0.0)	(0.0,0.0)	(0.0,0.0)	(0.0,0.0)	(0.0,0.0)	(0.0,0.0)	(0.0,0.0)	(0.0,0.0)	(0.0,0.0)
8-value (Interval)	(1.65,0.19)	(0.0,0.0)	(0.0,0.0)	(0.0,0.0)	(0.0,0.0)	(0.0,0.0)	(0.0,0.0)	(0.0,0.0)	(0.0,0.0)	(0.0,0.0)
2-value (Quantile)	(0.88,0.15)	(1.25,0.07)	(0.73,0.11)	(0.68,0.08)	(1.1,0.98)	(0.85,0.04)	(0.75,0.11)	(0.75,0.07)	(0.6,0.07)	(0.55,0.05)
3-value (Quantile)	(1.53,0.17)	(0.5,0.08)	(0.15,0.06)	(0.03,0.1)	(0.5,0.11)	(0.2,0.04)	(0.18,0.06)	(0.0,0.09)	(0.03,0.09)	(0.25,0.04)
4-value (Quantile)	(1.38,0.18)	(0.25,0.1)	(0.0,0.03)	(0.33,0.06)	(1.35,0.08)	(0.0,0.03)	(0.0,0.03)	(0.0,0.04)	(0.28,0.06)	(0.0,0.03)
5-value (Quantile)	(1.55,0.19)	(0.0,0.06)	(0.0,0.02)	(0.43,0.07)	(0.5,0.11)	(0.0,0.01)	(0.0,0.02)	(0.0,0.04)	(0.4,0.07)	(0.0,0.01)
6-value (Quantile)	(1.56,0.20)	(0.0,0.0)	(0.0,0.0)	(0.0,0.0)	(0.0,0.0)	(0.0,0.0)	(0.0,0.0)	(0.0,0.0)	(0.0,0.0)	(0.0,0.0)
7-value (Quantile)	(1.78,0.19)	(0.0,0.0)	(0.0,0.0)	(0.0,0.0)	(0.0,0.0)	(0.0,0.0)	(0.0,0.0)	(0.0,0.0)	(0.0,0.0)	(0.0,0.0)
8-value (Quantile)	(1.44,0.20)	(0.0,0.0)	(0.0,0.0)	(0.0,0.0)	(0.0,0.0)	(0.0,0.0)	(0.0,0.0)	(0.0,0.0)	(0.0,0.0)	(0.0,0.0)

**Table 7:** Learning efficiency comparison of different discretization values (Interval).

*N*-value	Methods									
	TPDA	*gs*	*hc*	*iamb*	*mmpc*	*rsmax*	*tabu*	*fastiamb*	*interiamb*	*mmhc*
6-value	18.861	0.599	0.167	0.611	0.459	0.59	0.212	0.493	0.678	0.489
5-value	19.644	0.692	0.208	0.897	0.463	0.693	0.246	0.652	0.880	0.484
4-value	18.75	0.641	0.257	0.551	0.495	0.659	0.313	0.253	0.571	0.51
3-value	29.124	0.728	0.336	0.416	0.518	0.749	0.409	0.398	0.449	0.543
2-value	13.624	1.332	0.601	0.795	0.563	1.349	0.730	0.474	0.84	0.616

**Table 8:** Learning efficiency comparison of different discretization values (Quantile).

*N*-value	Methods									
	TPDA	*gs*	*hc*	*iamb*	*mmpc*	*rsmax*	*tabu*	*fastiamb*	*interiamb*	*mmhc*
5-value	16.924	0.79	0.206	0.887	0.575	0.782	0.255	0.318	0.97	0.588
4-value	16.282	1.077	0.252	1.065	0.576	1.07	0.325	0.248	1.211	0.583
3-value	15.083	1.125	0.321	1.357	0.533	1.125	0.398	0.384	1.944	0.582
2-value	11.109	1.462	0.613	0.814	0.563	1.463	0.739	0.431	0.925	0.666
